# A software tool to support follow-up care in a French childhood cancer cohort: construction and feasibility

**DOI:** 10.1186/s12885-024-11857-y

**Published:** 2024-01-25

**Authors:** Charlotte Demoor-Goldschmidt, Pascal Veillon, Maxime Esvan, Mathilde Leonard, Sophie Chauvet, Amandine Bertrand, Liana Carausu, Fanny Delehaye, Julien Lejeune, Jérémie Rouger, Pascale Schneider, Caroline Thomas, Frédéric Millot, Line Claude, Julie Leseur, Fernand Missohou, Stéphane Supiot, Nathalie Bihannic, Isabelle Debroise, Carole Jeanneaud, Esther Lebreton, Marianne Roumy, Les Aguerris, Jean-Marie Chrétien, Virginie Gandemer, Isabelle Pellier

**Affiliations:** 1 Department of Oncohematopediatrics, University Hospital of Angers, University of Angers, Angers, France; 2 Department of Radiotherapy, Centre François Baclesse, University of Caen, Caen, France; 3 Department of Supportive Care, Centre François Baclesse, University of Caen, Caen, France; 4 Inserm U 1018, Epidemiology of Radiation, Gustave Roussy, Villejuif, France; 5 Department of Biostatitics, University Hospital of Rennes, Rennes, France; 6 Department of Oncohematopediatrics, University Hospital of Nantes, Nantes, France; 7 Department of Oncohematopediatrics, IHope, Lyon, France; 8 Department of Oncohematopediatrics, University Hospital of Brest, Brest, France; 9 Department of Oncohematopediatrics, University Hospital of Caen, Caen, France; 10 Department of Oncohematopediatrics, University Hospital of Tours, Tours, France; 11 Department of Oncohematopediatrics, University Hospital of Rouen, Rouen, France; 12 Department of Oncohematopediatrics, University Hospital of Poitiers, Poitiers, France; 13 Department of Radiotherapy, Centre Leon Berard, Lyon, France; 14 Department of Radiotherapy, Centre Eugène Marquis, Rennes, France; 15 Department of Radiotherapy, Institut de Cancérologie de L’Ouest, Nantes, France; 16 Association Les Aguerris, Paris, France; 17 Data Science Department, Clinical and Innovation Direction, CHU Angers, Angers, France; 18 Department of Oncohematopediatrics, University Hospital of Rennes, Rennes, France

**Keywords:** Database, Medical tool, Personalized survivorship care plan, Childhood cancer, Adolescent cancer, Survivorship, Radiotherapy, Chemotherapy

## Abstract

**Background:**

Treatment summaries and a personalized survivorship care plans based on internationally approved, organ-specific follow-up care recommendations are essential in preserving the health and quality of life for cancer survivors. Cohorts made up of survivors of childhood cancer have made significant contributions to the understanding of early mortality, somatic late complications, and psychosocial outcomes among former patients. New treatment protocols are needed to enhance survival and reduce the potential risk and severity of late effects, and working with treatment databases is crucial in doing so.

**Construction and content:**

In the GOCE (Grand Ouest Cancer de l’Enfant [Western Region Childhood Cancer]) network, in a participative approach, we developed the LOG-after medical tool, on which health data are registered and can be extracted for analysis. Its name emphasizes the tool’s goal, referring to ‘logiciel’ (the French word for software) that focuses on the period “after” the acute phase. This tool is hosted on a certified health data server. Several interfaces have been developed that can be used depending on the user’s profile. Here we present this innovative co-constructed tool that takes national aspects into account, including the results of the feasibility/satisfaction study and its perspective.

**Utility and discussion:**

The database contains data relating to 2558 patients, with samples from 1702 of these (66.54%) being held in a tumor bank. The average year in which treatment started was 2015 (ranging from December 1967 to November 2022: 118 patients were treated before 2012 and registered retrospectively when seen in long-term follow-up consultations or for another cancer since November 2021). A short questionnaire was distributed to healthcare professionals using the tool (physicians and research associates or technicians, *n* = 14), of whom 11 answered and were all satisfied. Access to the patient interface is currently open to 124 former patients. This was initially offered to 30 former patients who were over 15 years old, affected by the disease within the last 5 years, and had agreed to test it. Their opinions were collected by their doctor by e-mail, telephone, or during a consultation in an open-ended question and a non-directive interview. All patients were satisfied with the tool, with interest in testing it in the long term. Some former patients found that the tool provided them with some ease of mind; one, for instance, commented: "I feel lighter. I allow myself to forget. I know I will get a notification when the time comes."

**Conclusions:**

Freely available to all users, LOG-after: (1) provides help with determining personalized survivorship care plans for follow-up; (2) builds links with general practitioners; (3) empowers the patient; and (4) enables health data to be exported for analysis.

Database URL for presentation: https://youtu.be/2Ga64iausJE

## Background

The interregional group GOCE (Grand Ouest Cancers de l’Enfant [Western Region of Childhood Cancer]), created in 2009 and recognized by the INCa (Institut National Du Cancer [French National Institute of Cancer]) in 2010, brings together the pediatric oncology units of 7 University Hospitals (Angers, Brest, Nantes, Rennes, Tours, Caen, and Rouen—though initially Poitiers) and 3 Cancer Centers (Eugène Marquis in Rennes, Western Institute of Oncology in Nantes, Centre François Baclesse in Caen). GOCE specializes in the care involved in childhood cancer. When it comes to a pathology as rare as childhood cancer (about 400 cases/year for the western region), the organization of quality research requires the creation of structuring tools and the pooling of available resources. Its primary objective is to improve the care and quality of life of children and young adults with cancer, through improved care, research, and teaching. GOCE was created in the early 2000s when several pediatric oncology teams in western France realized that none of them possessed all the skills required to provide the best care for children. The idea was to pool available capacities in order to offer maximum human and material resources to improve the quality of care in a constantly evolving field. It was felt that expert care paths needed to be defined, and that teams needed to work together, discussing patients collegially via a multidisciplinary consultation meeting. We set out, therefore, to form a collaborative group in which the knowledge of all could be employed towards a common goal, namely effective treatment as close to home as possible for all children in the region. Its ultimate aim is to guarantee all childhood cancer patients equal access to quality cancer care, regardless of their location. The group grew steadily until 2010, when it became an organization recognized by the French National Cancer Institute.

To meet our objectives, GOCE has created a prospective cohort (ReCaPGO, Recueil des Cancers Pédiatriques du Grand Ouest [Data Collection of Pediatric Cancers in Western France]) of patients who were treated when they were under 25 years of age and were managed by the pediatric oncology teams within GOCE. This database was built around 5 modules: (i) identity module; (ii) a module recording the clinical information available at the time of diagnosis and at the beginning of the treatment; (iii) a module recording the summary of the interregional multidisciplinary staff and the suggested treatment planning; (iv) a module corresponding to information related to the tumor biobank of the West France region; and (v) a module intended to group together the elements of long-term follow-up (LTFU), both medical and psycho-social. This collaborative cohort was initiated to optimize patient care and research. The database was built as a lever for harmonizing the medium-term follow-up of patients in the region, for developing research projects, and, depending on the results, to be able to propose care recommendations (whether for acute care, supportive care, or follow-up). Given the vast improvement in long-term survival after childhood cancer over recent decades and the developments in knowledge of secondary chronic health diseases—and, of course, the observation that this follow-up could not be carried out in its entirety by general practitioners—it is clear that dedicated care is needed [1–9]. Initial (0 to 5 years after the treatment) and long-term follow-up (over 5 years after the treatment) should offer optimal and standardized care for patients based on current guidelines and recommendations [6, 10–12]. Personalized Survivorship Care Plans (PSCPs) have been promoted as a way to provide survivors and providers with information and resources to engage in risk-based, cancer-related follow-up care in multiple care settings [13, 14]. Within this framework, we have migrated from a simple searchable database to the addition of an entire interface to assist in care with a module able to generate a personalized follow-up plan, a patient module to empower the patient with personalized information (which is different from a patient search on the web), and a link between hospital doctors and private practitioners. Tools that, like this, include an algorithm for personalized follow-up plans, have recently been shown to facilitate clinicians’ ability to follow guideline recommendations in clinical practice [15]. Nevertheless, the precise follow-up is sometimes lacking, particularly but not exclusively after modern radiotherapy treatment and innovative treatment, because to date we have only limited data concerning late sequelae, particularly among childhood cancer patients. To better analyze and improve our practices, it would be beneficial to standardize monitoring practices.

This paper sets out to describe the design, methodology, and data availability of LOG-after software and the first results coming from the feasibility study.

## Construction and content

### Study population

The ReCaPGO cohort was initially a prospective cohort, but its current cohort is still recruiting. This cohort registers the data from all patients since 2012 under 25 years of age whose treatment has been managed by the pediatric oncology teams in 11 centers of the inter-region GOCE for cancer, hematological malignancy, histiocytosis, or bone marrow aplasia and for whom consent has been obtained (including parental consent when the patient is a minor). Since 2021, patients whose treatment occurred in the past (before 2012 or between 2012 and 2021 but not registered) could be included retrospectively after being seen in a consultation in one of the centers. Former patients are invited to come back for specific LTFU consultations. They are identified using existing, prospectively kept patient registries, patient listings, and medical record archives from departments of hematology/pediatric oncology and radiation oncology.

### Data collection

**Fig. 1:**
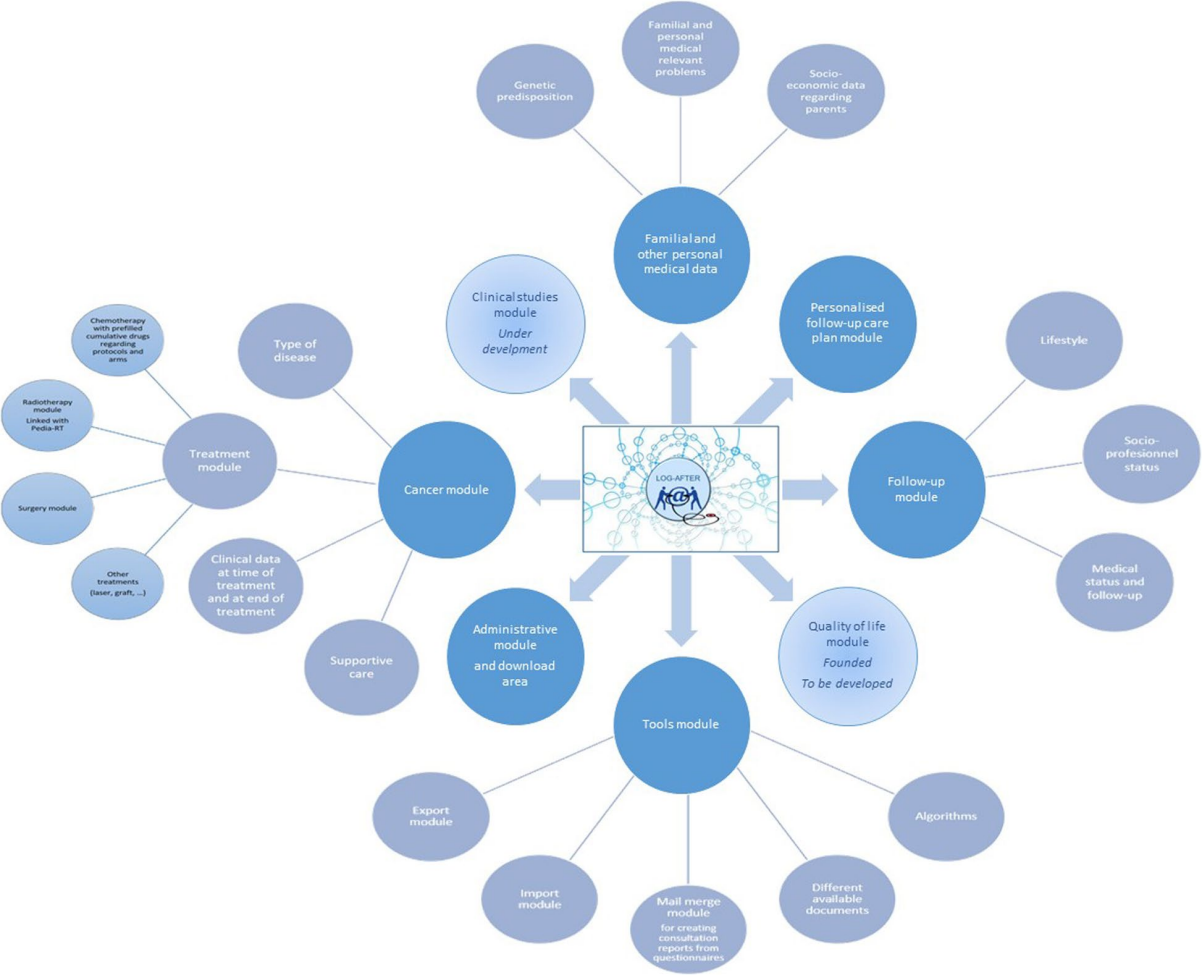
The different LOG-after software components—developed or planned

Legally, the LOG-after software is a medical software, which by definition includes a large amount of health data that is necessary for adapting the follow-up care of cancer. The different components of the software are presented in Fig. 1. Data on eligible childhood cancer survivors are entered without an anonymous identifier, but when data are extracted for studies, these data are pseudo-anonymized using generated identifiers. The software makes it possible for some patients to belong to several centers at once (because of the network of expertise, e.g., allograft, abdominal or neurological surgery, radiotherapy, proton therapy, center accredited for early phase research protocols, LTFU care, etc.), reflecting common practice in oncology and pediatrics and thus reducing the risk of duplication. Data on tumor type, date of diagnosis, and treatment were extracted from medical records, as well as personal and familial medical histories and administrative data (e.g., gender, date of birth, etc.). Treatment information includes details on surgery in terms of carcinological impact (complete resection, microscopically incomplete, macroscopically incomplete) and functional impact on the organ (partial or complete removal of the organ), radiotherapy (at least field, prescribed dose, dates), chemotherapy (protocol and arm of treatment, cumulative doses, dates), hematopoietic cell transplantation and the nature of the graft, and other supportive medication (e.g., corticotherapy, transfusions, fertility preservation). Chemotherapy information is abstracted from chemotherapy charts, medical records, and using an informative table that proposes the theoretical cumulative doses according to the different arms of the different chemotherapy protocols, thus limiting the potential margin for errors. These theoretical cumulative doses can be further adapted if necessary. In addition, some clinical information is added such as height and weight of the child at the time of the treatment and at the end, as well as social data (current class, sports practice, etc.). Data about follow-up and sequelae, including health behavior and demographic and socioeconomic characteristics, were deduced from physicians' reports or medical records. All of these data were entered into the database by trained data managers in the different centers or directly by the doctors themselves.

Participation in various studies was also collected, including the maintenance of a tumor bank. In the framework of certain studies (local or national), self-questionnaires (on, e.g., experience with the screening of second tumors in the framework of the DeNaCaPST study [16]) are given to the patient, which can be answered directly in the software via a dedicated interface for the patient, or in the near future (as in the case of the follow-up of patients after hematological pathologies, LEA study [17]).

### Linkage with other registries

The first input of data was from a regional database, the ReCapGo database. Other linkages with other regional or national databases, as with the national register of Childhood Cancer, are under consideration to decrease the time taken to enter the data. A link has recently been established with the National Pediatric Radiotherapy Database, PediaRT, organized by the French Group of Pediatric Radiotherapy (GFRP). This database includes, prospectively, all the DICOM-RT (Digital Imaging and Communications in Medicine Standard–Radiotherapy) data collected since 2013 in the 19 French reference centers. Based on the dose-volume histograms for all organs, a module has been developed using LOG-after for exporting data in organizing the follow-up of centers. The automatic import module is currently being implemented.

MapInMed is another tool developed by the Inserm unit ANTICIPE “Cancer and Prevention” that has been connected with the LOG-after database to study the impact of socio-demographic inequalities on different endpoints. Geocoding patients’ residence addressed (from the name of road and city) in the database is currently underway based on an aggregate indicator of deprivation using several variables associated with objective and subjective poverty: the French EDI (“European Deprivation Index”), which provides a score calculated for each small geographical unit of the French territory (around 2000 inhabitants) named IRIS (Ilots Regroupés pour l’Information Statistique [Merged Islet for Statistical Information]). This score is categorized into quintiles according to their departmental distribution: quintile 1 corresponds to the most affluent areas and quintile 5 to the most deprived areas.

### Health outcomes and lifestyle factors

During follow-up, all health outcomes (physical and psychological) including chronic pain, chronic fatigue, and aesthetic sequelae, are registered using a grade when necessary (CTCAE v5 most of the time) and are classified in different categories related to the different organs. Registered data about lifestyle habits include: smoking status; cannabis use; unhealthy alcohol use; and physical activity.

### Evolution of the software: database becoming a medical tool

**Fig. 2:**
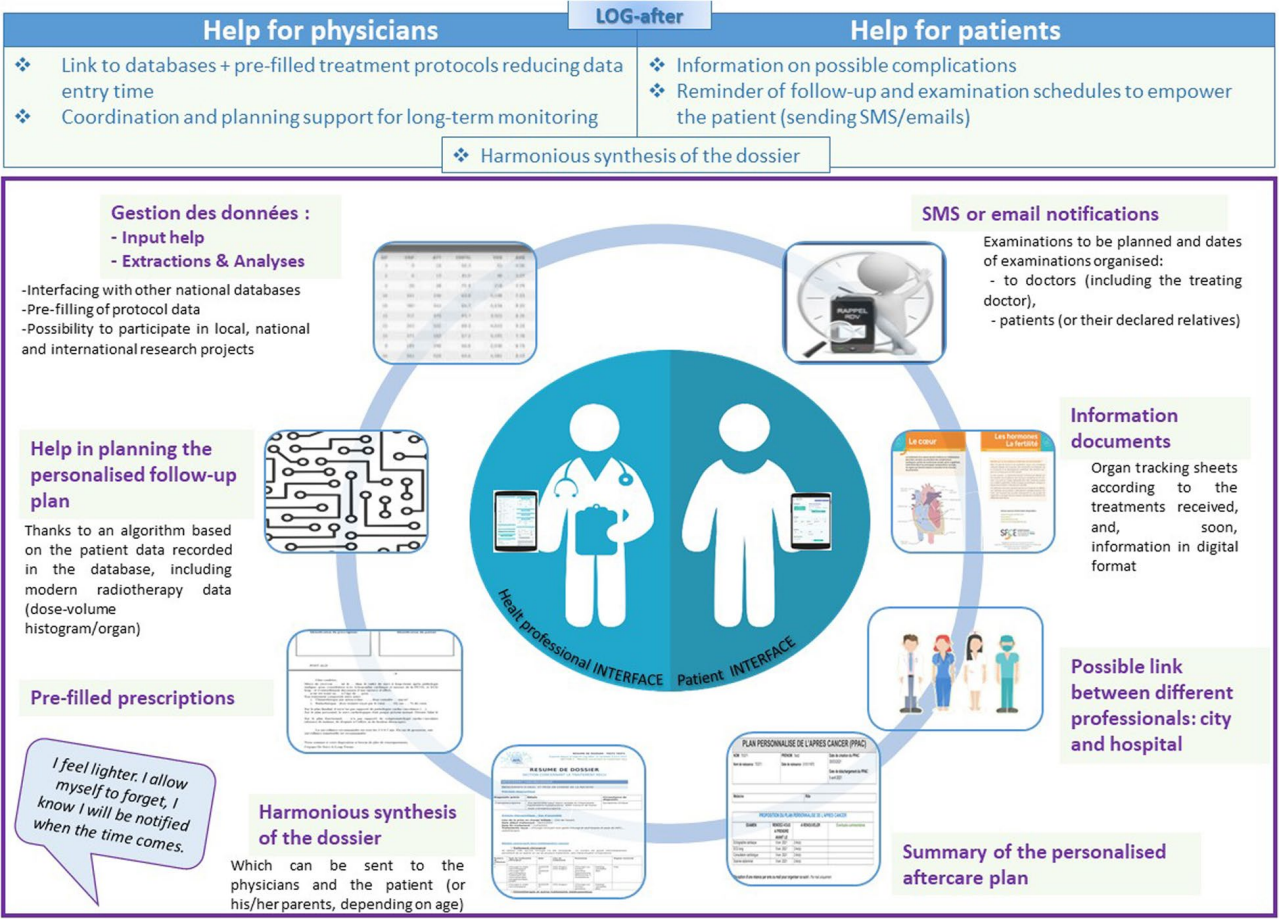
Figure summarizing the features of the LOG-after care software: a patient-centered tool for initial and long-term follow-up after a childhood or young adulthood cancer

- A: a tool for professionals (Figs. 1 and 2)

### Harmonious summary

Alongside the database, we developed an easily downloadable electronic module that delivers a summary of the patient’s oncological history (to be implemented in the patient’s medical record, or on the national shared medical record, for example).

### Help for the PSCP

Another module was developed that simplifies the provision of personalized care for follow-up after cancer. This module is based on an algorithmic approach taking into account data about the cancer, personal factors and existing comorbidities (constitutional mutation, family field, other personal medical history), eventual participation in research studies, and evidence-based guidelines. The algorithm also takes into account certain data recorded during follow-up (such as a possible thyroidectomy in a patient who has received cervical radiotherapy, which no longer justifies follow-up regarding the risk of a second tumor in this organ). This algorithm can be easily adapted and updated. The different guidelines that are in current use for initial follow-up and LTFU have been taking into account, such as those of the International Late Effects of Childhood Cancer Guideline Harmonization Group (IGHG), initiated in 2010, which establishes recommendations, through international collaboration, for the surveillance of late effects in childhood cancer survivors [3, 18]. In addition, as most of the IGHG recommendations are not very precise with regard to radiotherapy data, recommendations from the PENTEC group (Pediatric Normal Tissue Effects in the Clinic) were added [19]. In addition, the algorithm takes into account the accuracy of the recorded data. Where radiotherapy is concerned, for example, if dose data on given volumes concerning the different organs are available, the algorithm takes this information into account. But if there is only information on the field and the prescribed dose, the algorithm will work in degraded mode by specifying that the precise dosimetric data are missing. For example, if a supra-diaphragmatic radiotherapy was able to spare the mammary glands, the algorithm will not propose any breast monitoring (unless there is another risk factor). On the other hand, if no dosimetric data are provided, in the presence of this type of field, breast cancer surveillance will be proposed, specifying that the precise radiotherapy data are missing. As approximately 10% of children with cancer have an underlying cancer predisposition syndrome, the algorithm took this information into account too. The main source of data in this regard was the Childhood Cancer Predisposition Workshop Articles, a collection of 18 position papers stemming from the Childhood Cancer Predisposition Workshop Series held in October 2016 by the Pediatric Cancer Working Group and published in 2017 [20].

We also developed an algorithm based on published international guidelines where these existed. Where they were absent, however, we drew on national recommendations and expert consensus, This algorithm takes all of the information collected in the database into account to create individualized surveillance plans [2, 21]. The recommendations provided also took into account the possibilities of follow-up and management in clinical practice in France (among others, for the recommendations on fertility preservation or evaluation). Currently, 53 end points (including complementary examinations, specialized consultations, and educational tools) are covered by the algorithm and each end point includes between one and 34 conditions.

In practice, when the practitioner runs the algorithm, the software proposes a personalized follow-up plan and possible educational tools for the patient. It specifies for each recommendation the type of examination, the recommended or suggested frequency, the level of evidence related to this recommendation according to the rating system used in international guidelines for each recommendation, and the associated bibliography used to establish this condition [18]. It also offers notes for use by professionals and patients. These notes have been reviewed by an association of former patients to discern how best to inform patients in a way that can be easily comprehended. The physician can then deselect, modify, or add follow-ups. All modifications are traced so that they can be analyzed.

### Planning module

Once the personalized follow-up plan has been validated by the physician, a planning module is available. This module sends notifications to the patient and to the attending physician according to the planned frequency of the examinations and to identify those patients who are becoming less responsive to participating in follow-up. Breaks in the follow-up are possible (e.g., for pregnancy, intercurrent health events, long trips abroad), and it is also possible to permanently stop the planning (e.g., readjustment of the follow-up from initial follow-up to LTFU.

### Sharing data

Although the number of former patients is significant, the number of childhood and young adult cancer survivors per general practitioner (GP) is low (on average 1 to 2 patients per GP). Moreover, doctors are overloaded with emails, letters, and other documents that are time-consuming to read and file. In this context, with the patient’s agreement the LOG-after care interface can be shared with the GP and any other professionals involved in the patient’s follow-up in order to help link the GP with hospital-based clinicians for a coordinated, patient-centered follow-up. At any time, the patient can see who has access to his or her file on his or her own interface.

### Documents

Practitioners can also gain access to a range of documents, including pre-filled prescriptions, useful articles, clinical studies documents, and more.

- B: A tool for patients (Fig. 2)

Nowadays, several apps and web sites have been developed that support the personal management of chronic or long-term physical conditions for adolescents and adults treated for cancers [22–25] or for chronic disease [26]. The French association of former patients, “Les Aguerris”, also sought after a dedicated online application. Thus, in a participative approach including former patients and two national associations of former patients, “Les Aguerris” and “On est là”, we designed and developed a patient interface for the LOG-after software, through which patients can have all of their key health documents at their fingertips. This interface aimed to help at the time of transition (from initial follow-up to LTFU and from childhood to adulthood), to help the patient to become autonomous in his or her follow-up, and to empower the patient in taking responsibility for his or her healthcare. Using this interface, former patients can download a summary of their medical records and follow-up recommendations. They can also fill in self-questionnaires if they participate in research studies, receive notifications when it is the right time to schedule a follow-up according to the personalized follow-up plan set up with their doctor, follow the planning of their examinations, and access links to educational resources (whether videos^1^ of professional explanations and former patients testimonies, or texts) [27].

In the next few years, a patient module dedicated to quality of life and supportive care will be developed under a European project. It will function as a digital companion to meet patient needs.

## Data protection

The LOG-after software is an electronic medical record (EMR) software. Patients received a clear and comprehendible information about data collection and are free to agree or disagree to their inclusion. And 99.9% of the patients (all except one) for whom this EMR is used agreed that their data can be extracted and reused for research on collected data. An information letter, reviewed and validated by an ethics committee, is available and given to patients and/or legal authorities. Data protection complies with current local regulations: CNIL (Commission nationale de l’informatique et des libertés [National Commission for Information and Liberties]) and the software is hosted by an approved and secure health data server compliant with French regulations: HDS (Hébergement de Données de Santé). To analyze and explore the data collected, a consortium agreement has been signed between all the user centers, specifying the organization. In addition, a multi-professional steering committee representing all the centers meets quarterly to propose and validate important decisions concerning software upgrades and potential studies. The General Data Protection Regulation (GDPR) balances the right to privacy with exchange of data and all linkages formed followed the guidelines of the GDPR.

## Development under a feasability study

### Method

The tool was developed as described above. Experimentation with it in daily practice was subjected to a feasibility study, which ran from April 2021 to December 2022. A satisfaction questionnaire was sent to the participating professionals (invitation by e-mail to fill an online questionnaire). The patient interface was initially offered to 30 former patients from 3 centers who were over 15 years old had had been affected by the disease within the last 5 years, all of whom agreed to test it. Their opinions were collected by their doctors by e-mail, telephone, or during a consultation in an open-ended question and a non-directive interview.

### Results of the feasibility study

The development of the tool and its implementation in daily practice were included in a feasibility study, which was conducted from April 2021 to December 2022. A satisfaction questionnaire was sent to the professionals (*n* = 14), with 11 answering, all of whom were satisfied (all answers on a Likert scale were 3 or 4 out of 4). Some points of improvement were raised and, for the most part, they could be taken care of because they were minor. Indeed, as the software is based on a software package, we are able to adapt most of the points without going back to the computer developers. Others, such as minimizing the actions required to close a file in the event of death or to register a momentary pause in the follow-up due to a late relapse or serious intercurrent illness, will require dedicated development because they affect the very structure of the software. Access to the patient interface is currently open to 235 former patients. This was initially offered to 30 former patients, over 15 years of age, who had been affected by the disease within the last 5 years, all of whom agreed to test it. All patients were satisfied with the tool, and registered their interest in testing it in the long term. Some users found that the tool provided them with a degree of ease of mind; one, for instance, commented: "I feel lighter. I allow myself to forget. I know I will get a notification when the time comes."

## Current use and perspective

At present, the database contains the detailed data about the first cancers (diagnosis, treatment) of 2558 patients, including 2311 living patients, with tissues from 1702 of them (66.54%) held in a tumor bank. Not all modules have the same degree of completion and we first focused on the cancer treatment received (including supportive care), which is a necessary step in launching the follow-up module and its algorithm. The availability of biological material and information about inclusion in clinical studies or cohorts is also a very well completed module, permitting us to participate in different forms of analysis. The average year of treatment was 2015 (ranging from December 1967 to November 2022; 118 patients were treated before 2012 and registered retrospectively when seen in LTFU consultations or for another cancer since November 2021 (Table 1)). For 40 patients, a second cancer was registered (11 sarcomas, 10 cerebral tumors, 6 cases of leukemia, 5 of lymphoma, 5 adult-type cancers, 3 rare tumors), and for 4 patients, at least 3 cancers were registered. For 327 patients, second-line therapies were registered due to recurrence(s) (33.3% for cerebral tumor, 23.8% for leukemia, 15.9% for sarcoma) including 47 allografts (Table 1). With regard to treatment, 41.5% had surgery (of whom for 16.1% it was the only treatment), 97.1% had medical treatment (including chemotherapy, targeted therapy), 20.5% had radiotherapy, and 18.6% had both (Table 1).

**Table 1:** Profile of the cohort for whom all data are registered

Disease	Number (%)
Leukemia	724 (28.3)
Cerebral tumor	506 (19.8)
Lymphoma	436 (17.0)
Sarcoma	322 (12.0)
Kidney tumor	156 (6.1)
Neuroblastoma	153 (6.0)
Other	112 (4.4)
Rare tumor	106 (4.1)
Extra-cerebral germ cell tumor	43 (1.7)
Total	2558 (100.0)
Patients	
Mean age at diagnosis (years) [range]	8.34 [0.0–23.2]
Sex boy/girl, n (%)	1392/1166 (54.4/45.6)
Tumor collection	
Available tumor collection	1469 (57.4)
Treatment as First Line	
Surgery	1061 (41.5)
Chemotherapy and oncological drug treatment	2228 (97.1
High-dose chemotherapy and autotransplant	91 (3.6)
Radiotherapy	523 (20.5)
Allograft	98 (3.8)
Simple monitoring	97 (3.8)

The LOG-after tool (Figs. 1 and 2) is used as a dedicated electronic medical record by all GOCE centers and in daily care, particularly when treating those who have dedicated LTFU consultations. The initial positive feedback, its link with national forces, its potential for development, and its link with patients have attracted the interest of several other centers outside GOCE, which are currently testing the software. At present, a number of clinical studies are considering the possibilities of having their case report form (CRF) hosted in LOG-after to limit data collection time and, at the same time, enrich LOG-after, while taking into account all the regulatory procedures inherent in clinical research and the GDPR (Fig. 2).

## Discussion and conclusion

Among all the symptom management interventions that exist, digital (or "e-") health technology is an emerging topic. It is broadly defined as using technology to promote, prevent, treat, and maintain health, health care, and supportive care. The Childhood Cancer International Survivors Network, an advocacy network of Childhood, Adolescent and Young Adult Cancer Survivors (CAYACSs), asked for “full disclosure and sharing of medical history and potential risks to current and future health” for all CAYACSs. Nevertheless, the availability of a LTFU care, a personalized treatment summary, and a survivorship care plan for every childhood and young adult former patient is still not equal throughout Europe [28]. A number of digital tools have been described and developed across Europe in response to this challenge, including SurPass (an Italian initiative now available in 6 European countries) [23], and the Scandinavian tool (currently available in 2 countries) [29]. While some of these tools focus on quality of life digital tools [30], others develop lifestyle modules for preventive care [4].

The LOG-after cohort, which is still recruiting, has been prospectively including patients from 2012 to the present from several centers across western France for the past year, as well as former patients seen in LTFU consultations or for another cancer. Additionally, in the last year, former patients from other centers outside of the West France region have been registered. The available data include a combination of detailed individual-level data on diagnosis and treatment, including treatment data on recurrences and subsequent tumors and their treatments, other personal and familial medical information, social and educative data, and highly complete outcome data on personalized follow-up care plans and various long-term health outcomes.

The algorithm of the LOG-after cohort is unique as it includes data about not only the cancer and its treatment, but also constitutional mutations, comorbidities, and follow-up. The global tool is also unique and is linked with the national database of pediatric radiotherapies, PEDIA-RT.

The database initially created in GOCE for research is now used, in addition, as an aid to care with the LOG-after tool.^2^ The feasibility study was positive and several additional centers have requested its use. This tool, which was built in collaboration with a range of health care professionals and former patients, aims to promote and homogenize initial and long-term follow-up. Two satisfaction studies have been initiated and will start in the next months, one dedicated to LOG-after’s use among adolescent and young adult patients, and one for childhood cancer patients with a focus on patients who were treated for a brain tumor. In both, it will include two phases: one to describe the key performance indicators as part of a small-group study in the humanities and social sciences; and a large auto-questionnaire about satisfaction. An impact study, which has been funded, will also start in one year and concerns LTFU care. It will randomize two groups of patients, with and without the patient and GP interface to compare the adherence to LTFU recommendations In addition, a European project will develop a dedicated module for patients targeting adolescents and adults regarding the quality of life for patients in remission.

## Data Availability

The datasets used and/or analyzed for the current study are available from the corresponding author on reasonable request. Access to the LOG-after database is possible after preparing a concept proposal from an external researcher and approval from the LOG-after committee. A set-up data user agreement will then finalize the process.

## Abbreviations

CAYACSs: Childhood, Adolescent and Young Adult Cancer Survivors
CRF: Case Report Form
DICOM-RT: Digital Imaging and Communications in Medicine Standard – Radiotherapy
GDPR: General Data Protection Regulation
GFRP: French Group for Pediatric Radiotherapy
GOCE: Grand Ouest Cancers de l’Enfant; Western Region of Childhood Cancer
GP: General Practitioner
IGHG: International Late Effects of Childhood Cancer Guideline Harmonization Group
INCa: Institut National Du Cancer; French National Institute of Cancer
LTFU: Long-term follow-up
PENTEC: Pediatric Normal Tissue Effects in the Clinic
PSCP: Personalized Survivorship Care Plans

## Footnotes

1 E.g., https://vimeo.com/449230423

2 https://youtu.be/2Ga64iausJE
